# Selective Choice of the Efficient Carotenoid Antenna by a Xanthorhodopsin: Controlling Factors for Binding and Excitation Energy Transfer

**DOI:** 10.1021/jacsau.4c01243

**Published:** 2025-06-26

**Authors:** Ishita Das, Ariel Chazan, Jonathan R. Church, Shirley Larom, Rosa León, Patricia Gómez-Villegas, Daniela Bárcenas-Pérez, José Cheel, Michal Koblížek, Oded Béjà, Igor Schapiro, Mordechai Sheves

**Affiliations:** † Department of Molecular Chemistry and Materials Science, Weizmann Institute of Science, Rehovot 7610001, Israel; ‡ Faculty of Biology, Technion-Israel Institute of Technology, Haifa 3200003, Israel; § Fritz Haber Center for Molecular Dynamics Research Institute of Chemistry, The Hebrew University of Jerusalem, Jerusalem 9190401, Israel; ∥ Laboratory of Biochemistry and Molecular Biology, Faculty of Experimental Sciences, Marine International Campus of Excellence (CEIMAR), 16743 University of Huelva, Huelva 21071, Spain; ⊥ Centre Algatech, Institute of Microbiology, Novohradská, Třeboň 37981, Czech Republic; # Faculty of Science, University of South Bohemia, Branišovská 1760, 370 05 České Budějovice, Czech Republic; ∇ The Nancy and Stephen Grand Technion Energy Program (GTEP), Technion-Israel Institute of Technology, Haifa 3200003, Israel

**Keywords:** proton-pump rhodopsin, xanthorhodopsin, carotenoids, light-harvesting antenna

## Abstract

Despite extensive research on carotenoids and microbial rhodopsins in aquatic environments, a fundamental understanding of the binding requirements of carotenoids that serve as auxiliary light-harvesting antennas for rhodopsins is still lacking. Our recent discovery of 3-hydroxylated xanthophyll-binding proteorhodopsins and xanthorhodopsins prompted us to investigate the role of keto and hydroxy functional groups in carotenoid binding to rhodopsins and their influence on energy transfer to the retinal chromophore. In this study, we examined the binding of 12 carotenoids to rhodopsin Kin4B8 (a protein of the xanthorhodopsin family, GenBank: OP056329) and assessed the energy transfer between the carotenoid and the retinal chromophore. We found that 3-hydroxylated xanthophylls were the most effective light-harvesting antennas among the carotenoids studied. While 4-ketocarotenoids also bound to the protein, their energy transfer efficiency was significantly reduced. In contrast, the presence of a 4-hydroxy group or the substitution of the β-ionone ring by an ε-ionone ring completely prevented binding. Furthermore, mutagenesis studies of Kin4B8 suggest that specific residues play a key role in the selective binding of carotenoids. These findings provide valuable insights into the structural determinants of rhodopsin-carotenoid interactions, which may aid in predicting the recruitment of various carotenoid antennas by retinal proteins.

## Graphical abstract

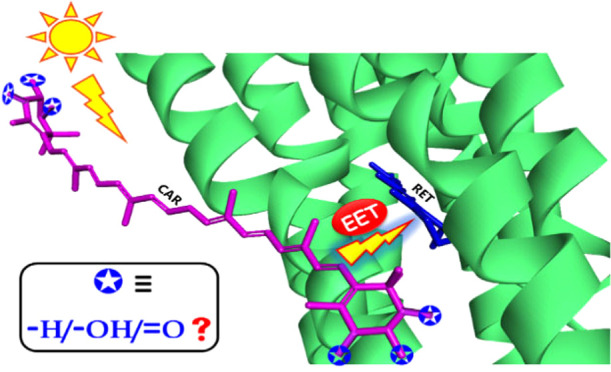

## 1 Introduction

Phototrophic microbes play a pivotal role in aquatic ecosystems by determining the amount of sunlight that is funneled to the microbial food web. One molecular system that facilitates the utilization of sunlight energy by phototrophic microbes is the rhodopsin protein, which uses a retinal molecule as the primary light-absorbing component to absorb light in the visible spectrum.^1,2^ Rhodopsin-bearing microbes are believed to significantly contribute to microbial phototrophy, as more than 50% of the bacteria in sunlit oceans possess a rhodopsin gene in their genome.^3^ Over the past two decades, it has become increasingly clear that many rhodopsins can bind an auxiliary antenna composed of a carotenoid, which boosts light absorption and enhances pigment activity. The bound carotenoid absorbs light in the blue-green spectral range (430–520 nm), which complements the spectral range of green-absorbing rhodopsins (520–550 nm). Thus, the carotenoid-bound complex enables the bacteria to harness light that is inaccessible without the carotenoid.^4^ Many aquatic bacteria possess a rhodopsin gene and can synthesize various carotenoids; yet, the correct pairing between rhodopsins and carotenoids remains poorly understood due to the lack of systematic investigation into the mechanistic binding requirements of these complexes.

Xanthorhodopsin (XR) from the halophilic bacteria *Salinibacter ruber* was the first identified rhodopsin proton-pump employing a noncovalently bound carotenoid (salinixanthin) as an auxiliary light-harvesting antenna.^5–8^ Later, in vitro studies demonstrated that *Gloeobacter* rhodopsin (GR) from *Gloeobacter violaceus* can bind diverse carotenoids (e.g., salinixanthin, canthaxanthin, and echinenone), and in vivo studies confirmed the functionality and increased efficiency of the carotenoid-bound complex.^9–13^ Further studies suggested a specific selection of XRs toward carotenoids, and mutation studies of carotenoid-GR interactions revealed strong carotenoid-rhodopsin binding motifs.^13^ Despite the abundance of diverse microbial rhodopsin-containing organisms, only a few rhodopsins have been identified to bind carotenoids.^14–17^ While the aforementioned rhodopsin, GR, and XR can bind 4-ketocarotenoids, a recent study indicated that the freshwater bacterium *Sphingomonas glacialis* AAP5 uses an XR bound to the 2,3,2′,3′-tetrahydroxylated xanthophyll nostoxanthin.^18^ Additionally, our recent findings revealed that the 3-hydroxylated xanthophylls, lutein and zeaxanthin, can form a complex with Kin4B8 (a member of the XR family), along with two other members of the proteorhodopsin (PR) family—KR1 (GenBank: BAN14807) from the marine flavobacterium *Dokdonia eikasta* and EINA29G6 (GenBank: UJI09384).^19^ These reports greatly enhance both the diversity and quantity of known rhodopsins that can bind carotenoids, as well as expand the range of known carotenoids that can serve as auxiliary antennas to rhodopsin proton pumps. Furthermore, we observed efficient carotenoid-to-retinal energy transfer, resulting in enhanced proton pumping in Kin4B8. The 3-hydroxylated xanthophylls transferred up to ∼ 50% of their excitation energy to the retinal chromophore of Kin4B8, a transfer efficiency comparable to that of the salinixanthin-XR complex. Interestingly, while salinixanthin (the native ketocarotenoid of XR) can bind to Kin4B8, it fails to serve as an effective energy-harvesting antenna. This finding underscores the importance of specific carotenoid-rhodopsin pairing for effective energy transfer and the need for a deeper understanding of the fundamental mechanistic binding requirements of carotenoids to rhodopsins.

In the present study, we investigate the role of carotenoids β-ionone functional groups (keto vs hydroxy) in controlling binding and energy transfer efficiency. Kin4B8 was chosen as the model rhodopsin, and a diverse set of carotenoids with variations in their β-ionone rings was selected to further explore both rhodopsin-carotenoid binding and energy transfer. Our results indicate that 3-hydroxylated xanthophylls are the most effective candidates for energy-harvesting antennae for Kin4B8. Mutation analysis of Kin4B8 identified the Ser208 residue as crucial for binding 3-hydroxycarotenoids.

## 2 Results and Discussion

### 2.1 Binding of the Carotenoids to Kin4B8

The binding of 12 carotenoids to Kin4B8 was monitored using absorption and circular dichroism (CD) spectroscopy (see Table 1). Kin4B8 was incubated overnight with the carotenoids and then purified using a Ni^2+^-NTA column (see Section 4). Carotenoid binding was indicated by the absorption bands of the carotenoids in the absorption spectrum. Further confirmation was obtained by CD spectroscopy. While Kin4B8 displayed a very weak positive CD signal at its absorption maximum, free carotenoids showed no discernible CD signal. Carotenoid binding to Kin4B8 resulted in the appearance of a sharp positive structured CD band near the carotenoid’s absorption maximum, accompanied by a negative peak around 550 nm. This bisignate CD signature of rhodopsin-bound carotenoids likely arises from the combination of induced chirality in the carotenoid molecule and exciton coupling between the retinal and carotenoid chromophores.^17,20–23^ Excitation energy transfer from the carotenoid to the retinal chromophore was examined by fluorescence excitation spectroscopy. All of the absorption, CD, and fluorescence excitation spectra of the protein-carotenoid complexes have been reported with the purified (Ni^2+^-NTA column purification) complexes only.

**Table 1:**
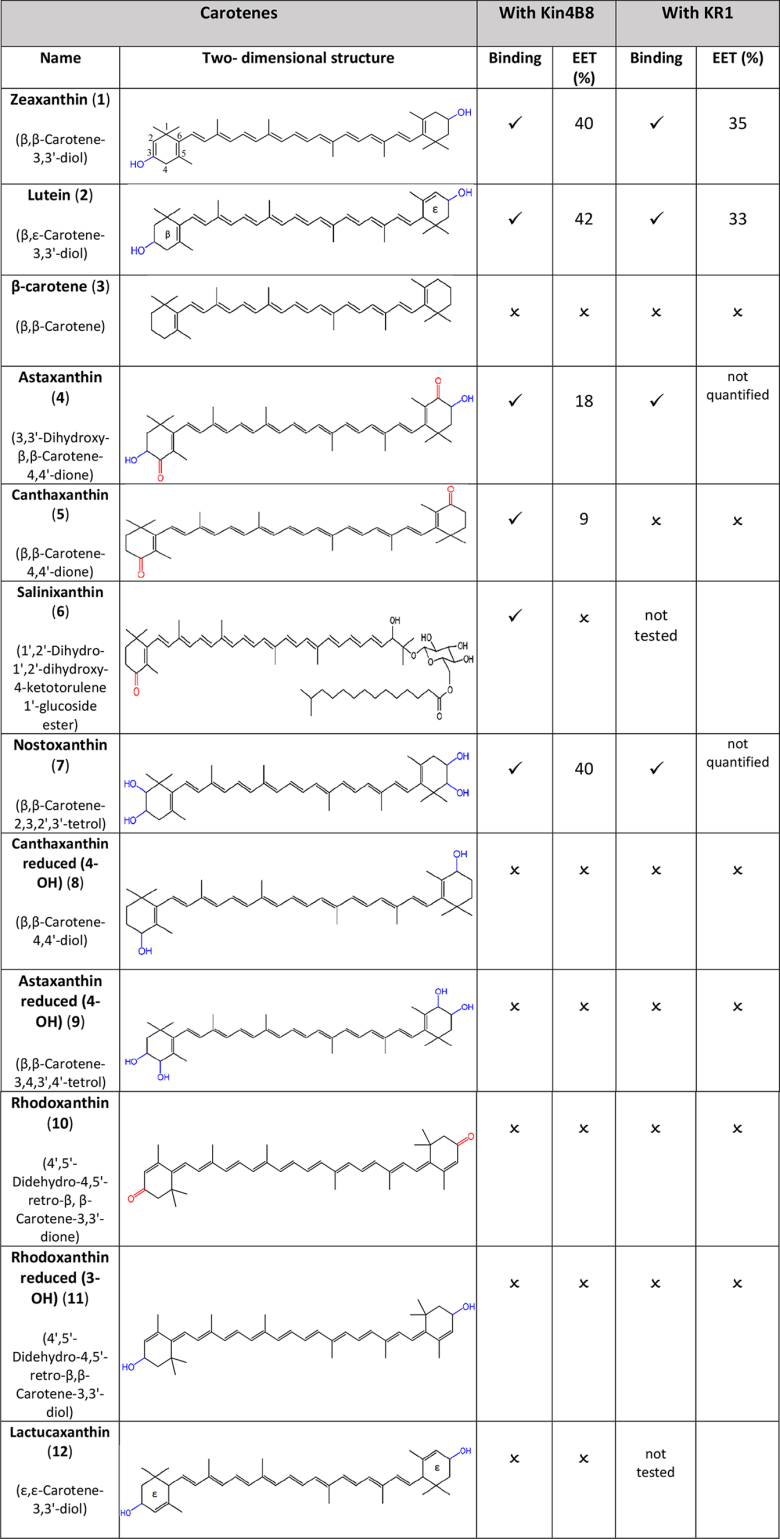
Summary of the Interaction of the Selected Carotenoids with Kin4B8 and KR1^a^ ^,^ ^b^

a Common and IUPAC semi-systematic recommended names are included.

b Error in calculation of EET is ± 5%.

#### 2.1.1 Zeaxanthin (1) and Lutein (2) [3-Hydroxy]

Zeaxanthin and lutein are structural isomers, differing only in the position of a single C=C bond on their ionone rings (see Table 1). In zeaxanthin, both rings are β-rings, while in lutein, one ring is β (C5=C6) and the other is ε (C4=C5). Our previous studies showed that both zeaxanthin and lutein efficiently bind to Kin4B8 and serve as light-harvesting antennae, each transferring about 50% of their excitation energy to the retinal chromophore.^19^

#### 2.1.2 β-Carotene (3) [No Hydroxy Group]

Unlike its 3,3′-dihydroxy derivative zeaxanthin, β-carotene does not bind to Kin4B8, as shown in our earlier work, highlighting the importance of the 3-hydroxy group for carotenoid binding.^19^

#### 2.1.3 Astaxanthin (4) [3-Hydroxy, 4-Keto]

Astaxanthin, which contains a 4-keto group in addition to the 3-hydroxy group, binds to Kin4B8 as indicated by absorption and CD spectroscopy (Figure 1a,b). However, its excitation energy transfer efficiency is ∼2.3 times lower (∼20%) than that of zeaxanthin (Figure 1c).

**Figure 1:**
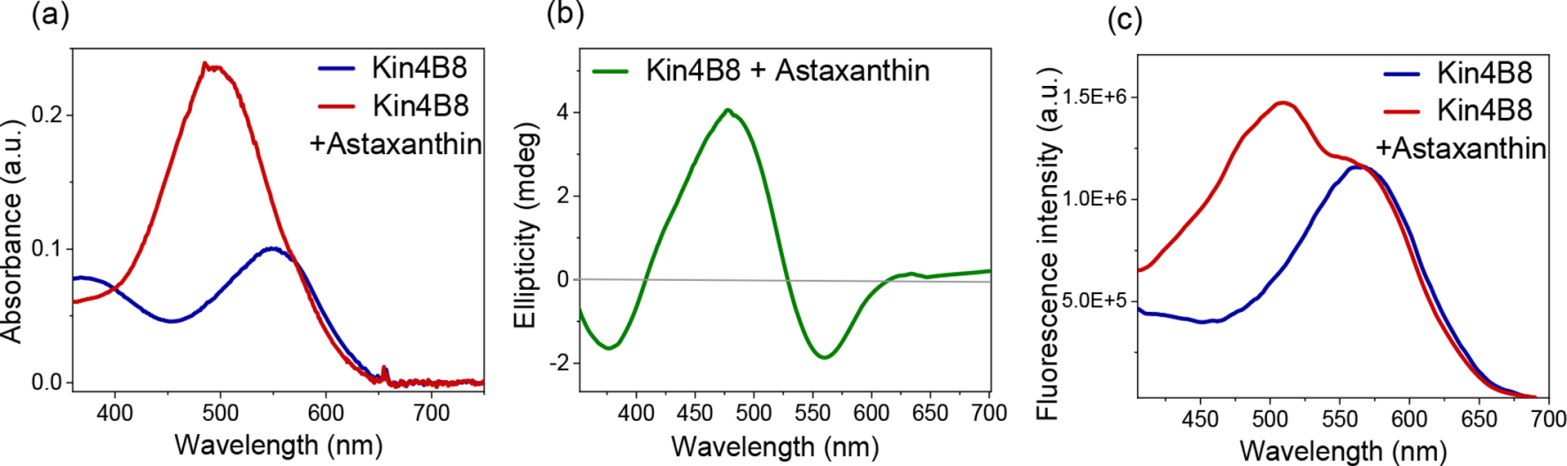
Interaction of astaxanthin with Kin4B8 studied with (a) absorption, (b) CD, and (c) fluorescence excitation spectroscopy.

#### 2.1.4 Canthaxanthin (5) [4-Keto]

In contrast to astaxanthin, canthaxanthin has only a single 4-keto group on the β-ionone ring. It binds to Kin4B8 (Figure 2), but energy transfer is reduced by approximately 4.5-fold compared to that of zeaxanthin.

**Figure 2:**
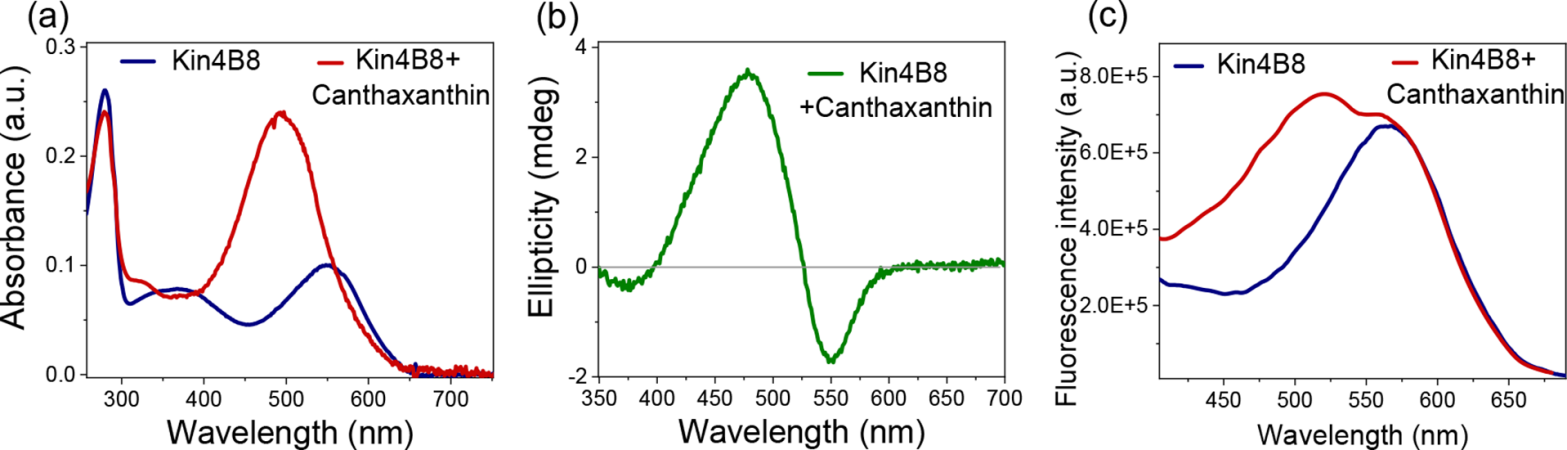
Interaction of canthaxanthin with Kin4B8 studied with (a) absorption, (b) CD, and (c) fluorescence excitation spectroscopy.

#### 2.1.5 Salinixanthin (6) [4-Keto]

Salinixanthin is the native carotenoid in XR from *Salinibacter ruber*. As it has been already reported previously, this 4-ketocarotenoid also binds to Kin4B8, but it does not transfer excitation energy to the retinal (Table 1).^19^

#### 2.1.6 Nostoxanthin (7) [2,3-Dihydroxy]

Nostoxanthin, which has an additional hydroxy group at C2, binds to Kin4B8 and transfers excitation energy with an efficiency similar to that of zeaxanthin (Figure 3, Table 1).

**Figure 3:**
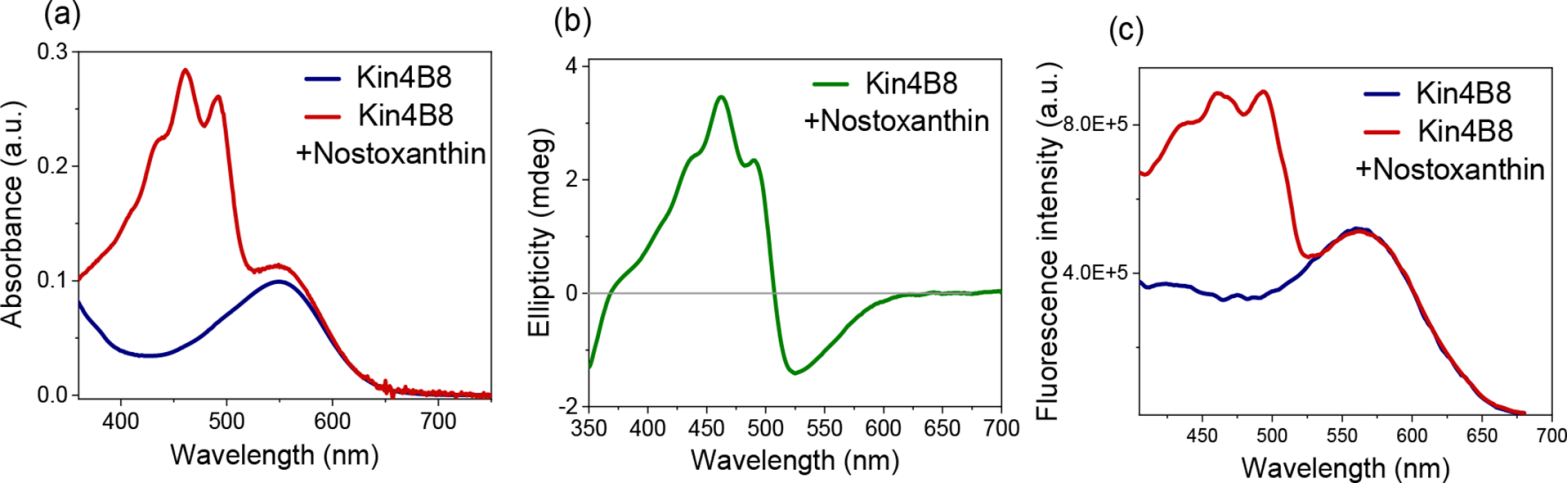
Interaction of nostoxanthin with Kin4B8 studied with (a) absorption, (b) CD, and (c) fluorescence excitation spectroscopy.

#### 2.1.7 Reduced Canthaxanthin (8) [4-Keto Reduced to Hydroxy]

The 4-keto group in salinixanthin is crucial for binding to XR and GR, as its reduction to a hydroxy group (salinixanthol) prevents binding.^9,24^ We reduced the 4-keto group in canthaxanthin to a hydroxy group using NaBH_4_. This modification completely disrupted its interaction with Kin4B8, as confirmed by absorption and CD spectroscopy (Figure 4).

**Figure 4:**
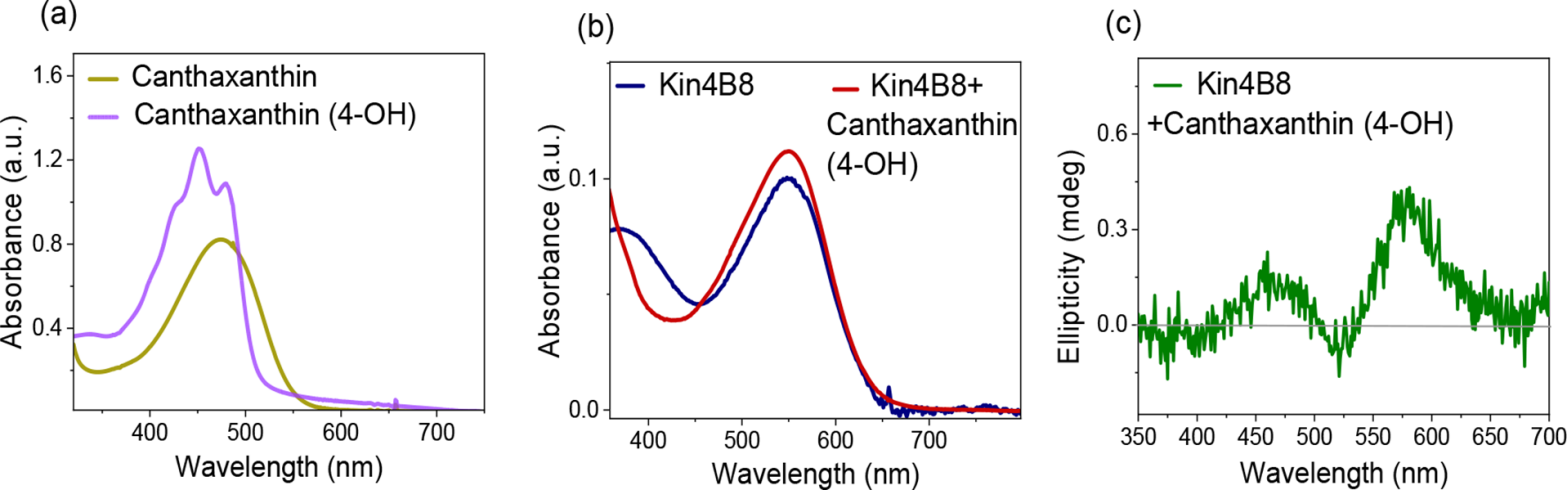
(a) Comparative absorption spectra of canthaxanthin and canthaxanthin (4-hydroxy) in ethanol. Interaction of canthaxanthin (4-hydroxy) with Kin4B8 was studied with (b) absorption and (c) CD spectroscopy.

#### 2.1.8 Reduced Astaxanthin (9) [3,4-Dihydroxy]

Following the results with reduced canthaxanthin, we tested the reduced form of astaxanthin (3,4-dihydroxy). Interestingly, its binding to Kin4B8 was significantly disrupted, and no typical CD spectrum of the bound carotenoid was observed (Figure S1), despite the presence of both 3-hydroxy and 4-hydroxy groups.

#### 2.1.9 Rhodoxanthin (10) [3-Keto] and Reduced Rhodoxanthin (11) [3-Hydroxy]

Rhodoxanthin contains a 3-keto group on the β-ring, and its ring-polyene adopts a planar conformation, unlike the twisted s-cis ring–chain conformation of zeaxanthin. Neither rhodoxanthin nor its reduced 3-hydroxy form binds to Kin4B8 (Figures 5 and S2), highlighting the importance of the ring–chain conformation for carotenoid binding.

**Figure 5:**
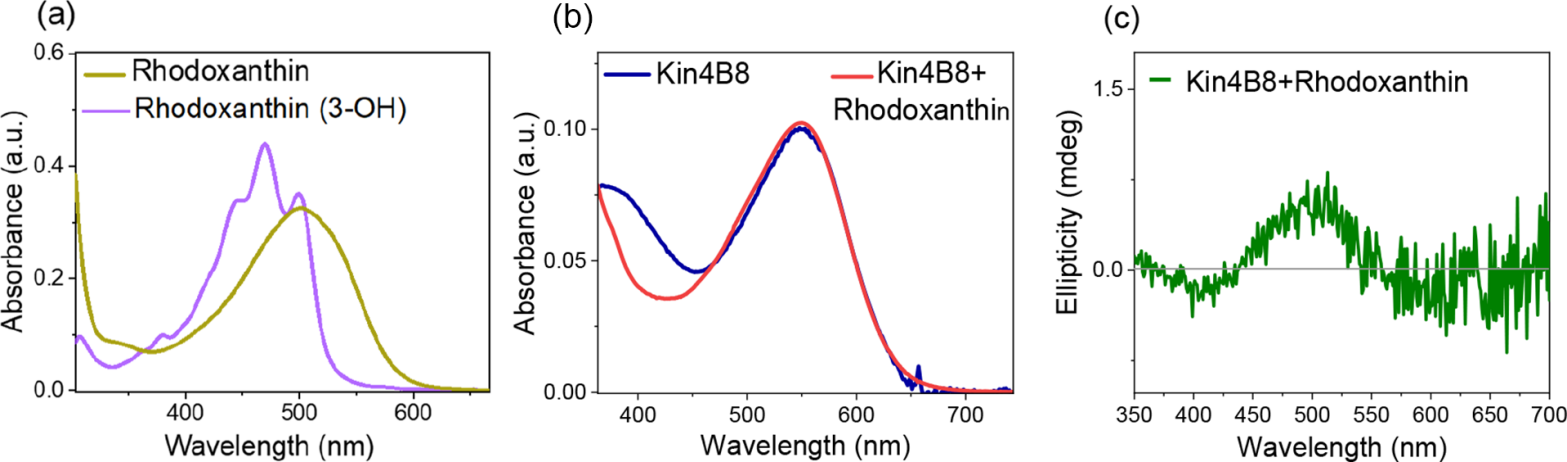
(a) Comparative absorption spectra of rhodoxanthin and rhodoxanthin (3-hydroxy) in ethanol. Interaction of rhodoxanthin with Kin4B8 studied with (b) absorption and (c) CD spectroscopy.

#### 2.1.10 Lactucaxanthin (12) [3-Hydroxy ε-Ionone Rings]

Lactucaxanthin, with two 3-hydroxy groups on the ε-ionone rings, does not bind to Kin4B8. The lack of conjugation between the ring’s double bond and the carotenoid polyene chain likely prevents its binding, as observed for rhodoxanthin and its hydroxy derivative (Figure 6).

**Figure 6:**
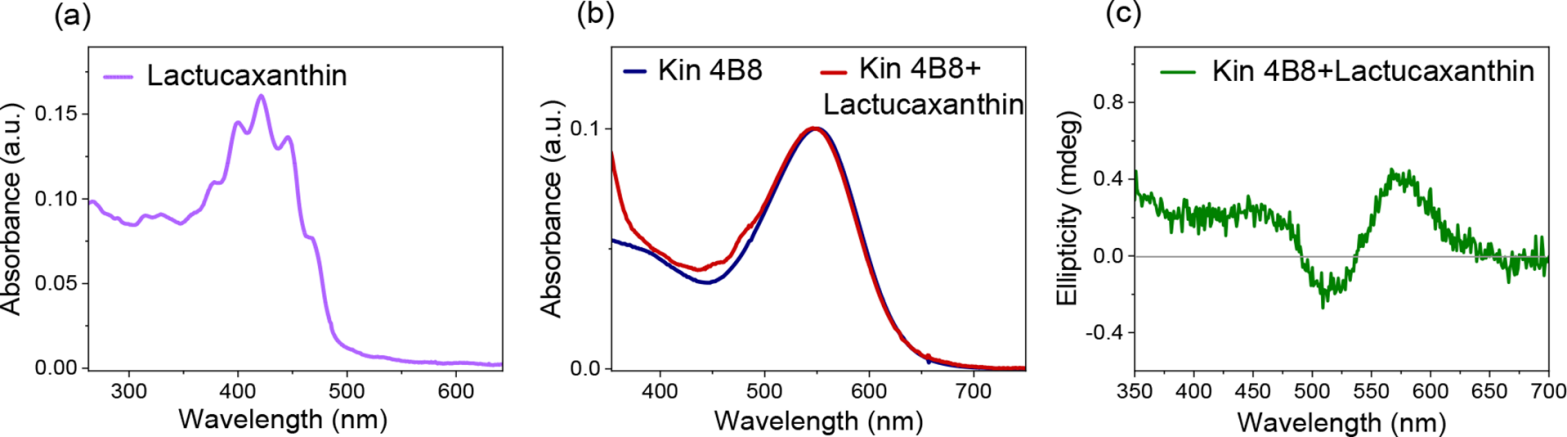
(a) Absorption spectrum of lactucaxanthin in ethanol. Interaction of lactucaxanthin with Kin4B8 was studied with (b) absorption and (c) CD spectroscopy.

The carotenoids screened in this study exhibit differences in their absorption spectra. Hydroxylated carotenoids, including zeaxanthin, nostoxanthin, lactucaxanthin, and the hydroxylated form of canthaxanthin (as well as astaxanthin and rhodoxanthin), display well-structured absorption bands, even in organic solvents (Figure S3). In contrast, the ketocarotenoids canthaxanthin, salinixanthin, astaxanthin, and rhodoxanthin show broader absorption spectra in organic solvents and lack the fine-structured absorption bands seen in hydroxylated carotenoids (Figure S4). It has been suggested that the fine-structured absorption in hydroxylated carotenoids reflects a defined twisted ring-polyene chain conformation due to rotation around the C6–C7 bond, which leads to a structured vibronic absorption band.^7,8^

Zeaxanthin (as well as nostoxanthin, Figure 3) bound to Kin4B8 retains its fine-structured absorption,^19^ and the same spectral pattern is also observed in the circular dichroism (CD) spectrum. This suggests that these carotenoids maintain a twisted 6-s-cis ring–chain conformation (Scheme S1) upon binding to Kin4B8. On the other hand, canthaxanthin and salinixanthin undergo a transformation from their broad absorption spectrum in organic solvents to a more structured band upon binding to GR or XR. This suggests that these ketocarotenoids adopt a twisted 6-s-cis ring–chain conformation when bound to GR or XR,^7,9^ a conformation that is further supported by the crystallographic structure of XR.^7,8^ In contrast, canthaxanthin and astaxanthin do not alter their broad absorption spectra when they are bound to Kin4B8 (Figures 2a and 1a, respectively). Furthermore, energy transfer from canthaxanthin to Kin4B8 is significantly less efficient than that observed in the zeaxanthin-Kin4B8 complex (Table 1), or as previously reported for the salinixanthin-XR/GR complex.^7,10,19^ Salinixanthin also does not exhibit a fine-structured spectrum upon binding to Kin4B8 and does not participate in excitation energy transfer.^19^ We suggest that the binding site in Kin4B8 is not suitable for accommodating the 4-keto-β-ionone ring in the s-cis twisted conformation of carotenoids such as salinixanthin, canthaxanthin, or astaxanthin. It is possible that the twisted s-cis ring–chain conformation of the carotenoid is necessary to establish a suitable dihedral angle between the transition dipole moments of the carotenoid and retinal polyenes, facilitating efficient energy transfer from the bound carotenoids to retinal. The binding efficiencies of the 4-ketocarotenoids are comparable to those of the 3-hydroxy carotenoids (with approximate binding percentages estimated and presented in Table S1). However, because the 4-ketocarotenoids do not adopt the ring–chain s-cis twisted conformation in the binding site, their energy transfer efficiency is poor.

Conversely, the Kin4B8 binding site appears to favor the s-cis twisted conformation of 3-hydroxy carotenoids, such as zeaxanthin, enabling efficient energy transfer. Interestingly, the two 4-hydroxylated (reduced) carotenoids, canthaxanthin and astaxanthin, do not bind to Kin4B8 (Table 1). In the XR or GR-salinixanthin system, it has been reported that salinixanthol (the 4-keto group of salinixanthin reduced to 4-hydroxy) no longer binds to the protein.^9,24^ Despite having a hydroxy group at the required C3 position, the presence of a 4-hydroxy group in astaxanthin prevents binding. The presence of a keto group at the 4-position of the carotenoid does not prevent binding but significantly reduces the energy transfer efficiency. In contrast, a 4-hydroxyl group completely abolishes the binding. We propose that the carbonyl group forms hydrogen bonds with Tyr and Ser residues near the carotenoid ring (Figure 7), facilitating binding. However, the energy transfer efficiency declines because interactions involving the keto group prevent the carotenoid from adopting the ring–chain s-cis conformation, which is crucial for establishing an optimal dihedral angle between the retinal and carotenoid transition dipole moments. The angle between these two chromophores in the Kin4B8-zeaxanthin complex, estimated from the EM structure (PDB ID: 8I2Z), was found to be 55°, similar to that observed in xanthorhodopsin.^7,19^ Conversely, the hydroxyl group at position 4 has a more pronounced effect, entirely preventing binding. This may be due to the hydroxyl group disrupting the hydrogen bonding network present in zeaxanthin. Additionally, modifications to the hydrogen bonding network of carotenoid ring B, involving residues Y191 and N188, may contribute further to this effect.

**Figure 7:**
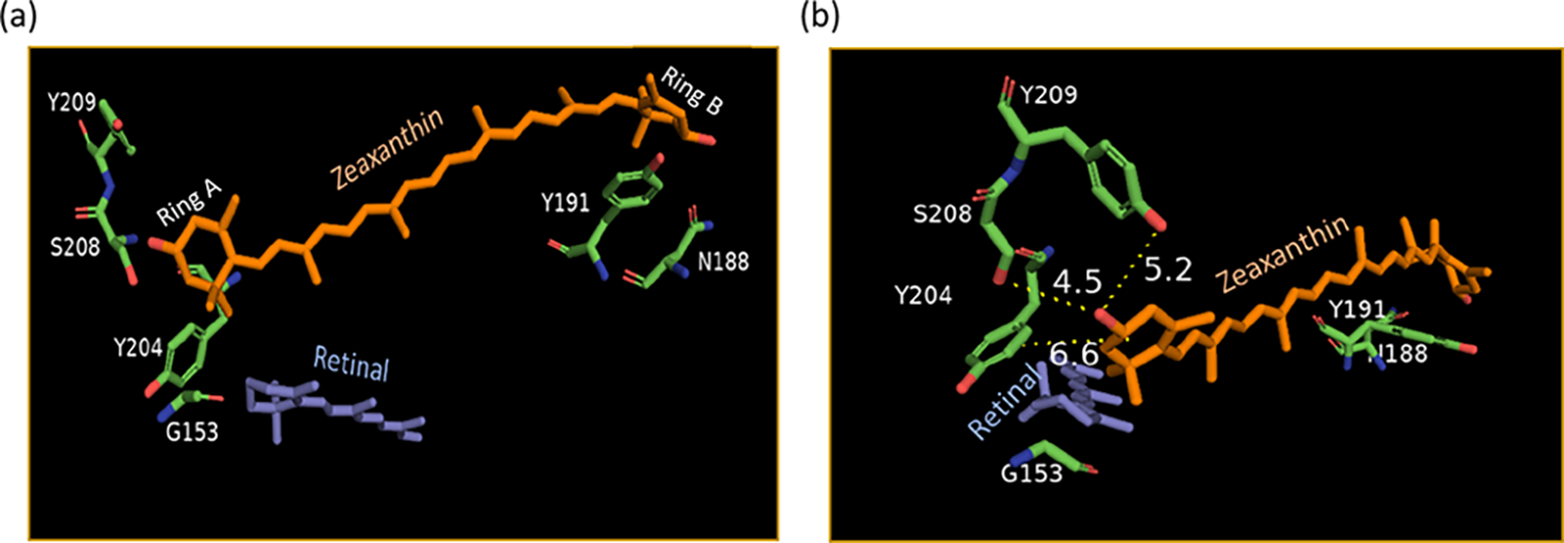
(a) Binding site of zeaxanthin in Kin4B8 (PDB ID: 8I2Z).^19^ (b) Interaction between the β-ionone ring (ring A-the ring close to the retinal β-ionone ring) ring of zeaxanthin and the polar residues in the proximity, the distances are marked that could possibly engage in hydrogen-bonding network. Y209-OH to −OH of the zeaxanthin ring distance: 5.2 Å; S208-OH to −OH of the zeaxanthin ring: 4.5 Å; retinal ring to the zeaxanthin ring distance: 6.6 Å.

The significance of the carotenoid chain-ring conformation was further demonstrated by the interaction of rhodoxanthin and its hydroxylated form with Kin4B8. Despite having the 3-hydroxy (or 3-keto) group, the planar ring–chain spatial arrangement of rhodoxanthin does not allow it to bind to Kin4B8.

The importance of the ring double bond location is illustrated by the study of lactucaxanthin. Lactucaxanthin has 3-hydroxy groups on both rings, but both are ε-rings, with the C=C bond located at C4–C5 instead of the C5–C6 bond found in zeaxanthin (both β-rings). This shift in the double bond position likely affects the conformation of the ring and its ability to bind to Kin4B8. Thus, even a minor alteration in the ring structure can prevent lactucaxanthin from binding to Kin4B8. Interestingly, lutein, which has one ε-ring and one β-ring, still binds efficiently to Kin4B8. Therefore, at least one β-ring appears to be a prerequisite for efficient interaction and binding. To quantify the effect of the double bond shift on the ring conformation, we have compared the geometries of the β-ring of zeaxanthin to the ε-ring of lutein from quantum chemical simulations. We observed a change of the dihedral C5–C6–C7–C8 from −56° (β-ring of zeaxanthin) to −124° (ε-ring of lutein) (Figure S8 and Table S2). The rotation by 68 degrees is due to a change from the sp^2^-hybridized C6 to a sp^3^-hybridized carbon, respectively.

Therefore, the functional groups and their position on the carotenoid rings as well as the ring–chain spatial conformation are pivotal in governing the energy transfer efficiency and the binding efficacy.

The absorption of the retinal chromophore remains unchanged upon carotenoid binding. Retinal absorption is sensitive to twisting around its single and double bonds, interactions with charges and dipoles, and the hydrogen bond network, particularly around the retinal protonated Schiff base. Therefore, it is plausible that carotenoid binding does not alter the retinal conformation.

### 2.2 Binding of the Xanthophylls to KR1

We screened several selected carotenoids (listed in Table 1) with the marine rhodopsin KR1 from flavobacterium *D. eikasta*, which belongs to the PR family and shares a similar carotenoid-binding site with Kin4B8.^19^ Similar observations to those made for Kin4B8 were obtained for the tested carotenoids, except canthaxanthin. Canthaxanthin did not bind to KR1, as evidenced by the CD spectrum (Figure S5). However, astaxanthin, which contains a 4-keto group and an additional 3-hydroxy group, still binds to KR1 and is involved in energy transfer, as shown in Figure S6. A summary of these results is provided in Table 1. It is possible that the hydrogen bonding network with the 4-keto ring of 4-ketocarotenoids is not compatible with the KR1 binding site. The replacement of the tyrosine residue (equivalent to position Tyr209 in Kin4B8) by aspartic acid in KR1 could be a contributing factor.

### 2.3 Potential Amino Acid Residues That Play a Role in Carotenoid-Kin4B8 Interactions

Several amino acid residues located near the zeaxanthin binding site in Kin4B8 can be identified from its cryo-EM structure.^19^ The carotenoid binding site is shown in Figure 7, based on the cryo-EM structure of the zeaxanthin-Kin4B8 complex (PDB ID: 8I2Z). An important residue in this context is Gly153, which is the equivalent of Gly156 in XR. Replacing this residue with a bulkier one prevents zeaxanthin binding to the protein.^19^ Two other residues, Ser208 and Tyr209, are situated near the carotenoid ring (Figure 7) and may play a role in protein-carotenoid interactions, potentially forming a hydrogen bonding network with the 3-hydroxy group of zeaxanthin. Tyr204 could also be key for binding and facilitating energy transfer, as this tyrosine is conserved among all known carotenoid-binding rhodopsins (XR, GR, Kin4B8, KR1, EinA29G6, etc.).

Based on this information, three mutants of Kin4B8—Y204A, S208A, and Y209F were generated for our study. In the S208A mutant, the polar serine was replaced with alanine, which disrupted zeaxanthin binding, reducing the binding efficiency to 2% of that of the wild-type Kin4B8-zeaxanthin pair, and no energy transfer was detected (Figure 8). The Y209F mutation, which eliminates the potential for hydrogen bonding but maintains the aromatic core, still allows zeaxanthin binding, as indicated by absorption and CD spectra. However, the energy transfer efficiency from zeaxanthin to retinal was reduced from ∼ 40% in the wild type to ∼18% in Y209F (Figure 9). The Y204A mutant was highly unstable and denatured during the protein purification process, preventing further analysis.

**Figure 8:**
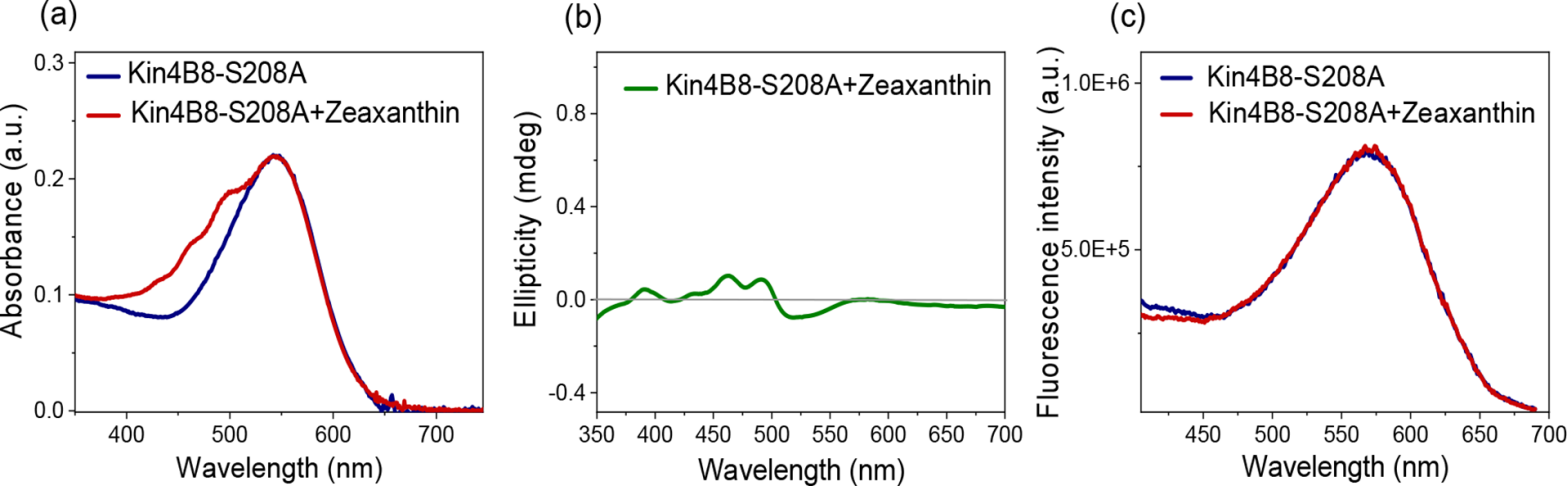
Interaction of zeaxanthin with Kin4B8–S208A studied with (a) absorption, (b) CD (c) fluorescence excitation spectroscopy.

**Figure 9:**
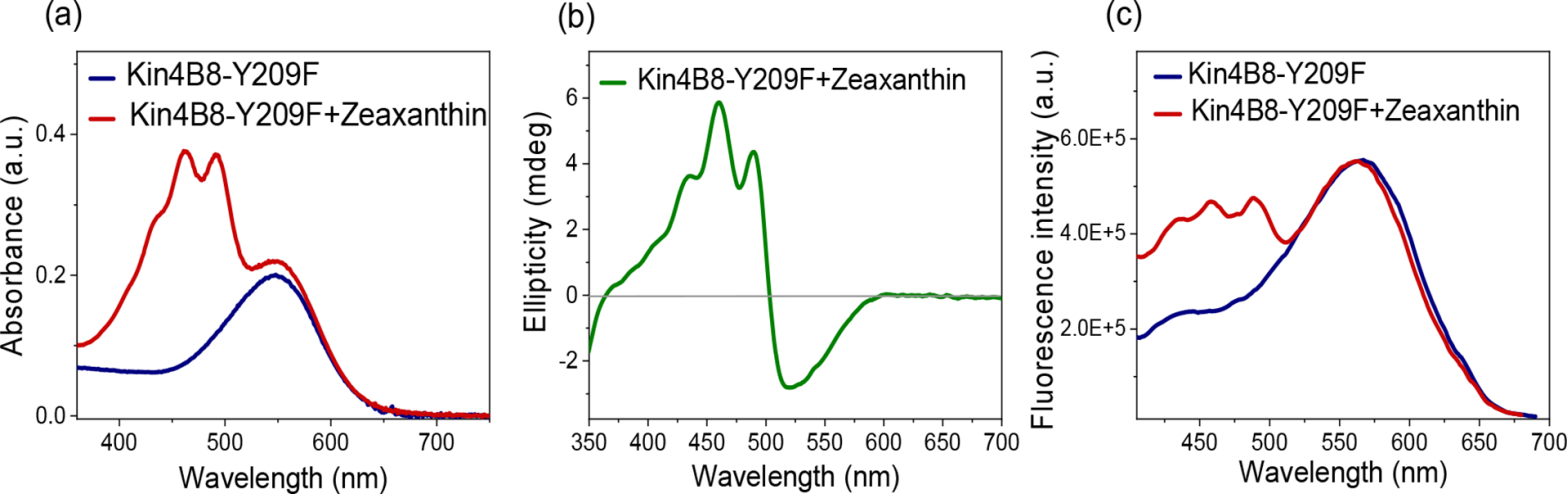
Interaction of zeaxanthin with Kin4B8–Y209F studied with (a) absorption, (b) CD, and (c) fluorescence excitation spectroscopy.

The results from the S208A and Y209F mutants strongly suggest that these residues are involved in hydrogen bonding with the 3-hydroxy group of zeaxanthin, stabilizing its binding and facilitating an efficient excitation transfer. Ser208 is crucial for zeaxanthin binding, while the hydroxy group of Tyr209 is likely essential for locking the planes of the zeaxanthin-retinal dihedral angle, which enables efficient energy transfer.

XR and GR utilize 4-ketocarotenoids as energy-harvesting chromophore antennas,^7–10^ in contrast to Kin4B8 and KR1, which bind 3-hydroxylated xanthophylls. This raises the question of which specific residues might determine the preferential binding of keto- or hydroxycarotenoids close to the retinal chromophore. A major difference between the carotenoid binding sites of XR and Kin4B8 lies at the bottom of the binding site. The Kin4B8 binding site contains two polar residues, Ser208 and Tyr209, near the hydroxy ring of zeaxanthin (Figure 7). Similarly, the homologous residues in KR1 (Thr and Asp) and EinA29G6 (Thr and Glu) are polar and can participate in hydrogen bonding with the 3-hydroxy group (Table 2). In contrast, the homologous residues in XR are hydrophobic (Met and Ala). Thus, the inhibition of binding 4-hydroxylated carotenoids like canthaxanthin (or astaxanthin) to Kin4B8/KR1 may be due to the disruption of the hydrogen bonding network in the presence of the 4-hydroxy group.

**Table 2:** Residues Close to Zeaxanthin as Obtained from the Cryo-EM Structure of Kin4B8-Zeaxanthin Complex (PDB ID: 8I2Z), along with Homologous Residues in XR, GR, KR1, and EinA29G6

	residue in TM6			
rhodopsin	204	205	208	209
Kin4B8	Y	A	S	Y
XR	Y	M	M	A
GR	Y	L	M	L
KR1	Y	M	T	D
EinA26G6	Y	I	T	E

All of the studied carotenoids have a symmetrical structure, except for lutein and salinixanthin. The fact that lutein binds and transfers energy as efficiently as zeaxanthin, in contrast to lactucaxanthin (Table 1), suggests that ring A (Figure 7a) of the carotenoid plays a dominant role in both binding and energy transfer. As discussed above, the interaction between the carotenoid and protein residues Y209, S208, Y204, and G153 (Figure 7) near ring A may be crucial for these processes. Ring B of the carotenoids can interact with polar residues Y191 and N188 (Figure 7); however, these interactions likely have a lesser impact on binding efficiency, as salinixanthin, which completely lacks a B-ring, still binds to KinRB8. This assumption is further supported by computational studies described below. Future research should further clarify the role of carotenoid ring B in binding and energy transfer to the retinal.

### 2.4 Computational Study on Carotenoid-Kin4B8 Interactions

To gain further insight into the factors that affect the binding of carotenes, we have performed hybrid quantum mechanics/molecular mechanics (QM/MM) simulations. For this purpose, we have selected three representative carotenoids with different structures: zeaxanthin, canthaxanthin, and 4-OH canthaxanthin. QM/MM molecular dynamics simulations (Figure 10) show that Ring-A of the zeaxanthin carotenoid (Figure 7) forms hydrogen bonds with both residues Ser208 and Tyr209. Once the interaction with Ser208 is weakened, the interaction with Tyr209 increases, and vice versa (Figure 10). This is in good agreement with the experimental observation that both residues are important for the binding, as mutating them decreases the binding of zeaxanthin. In contrast, canthaxanthin (4-keto) and (4-hydroxy) both form hydrogen bonds with Tyr209 exclusively. During dynamics, Ring-B (Figure 10) of canthaxanthin (4-hydroxy) is unable to form a hydrogen bond with either Asn188 or Tyr191. In contrast to both the 4-keto form of canthaxanthin and zeaxanthin, which each have a hydrogen bonding partner for the B-ring (Figure 10). The loss of this hydrogen bonding partner would destabilize the carotenoid over longer time scales and may help explain why a stable structure is not experimentally observed. Hence, ring A has less interaction with canthaxanthin than with zeaxanthin. In addition, the interaction of ring B decreases for 4-hydroxy canthaxanthin, which makes it overall unfavorable for binding.

**Figure 10:**
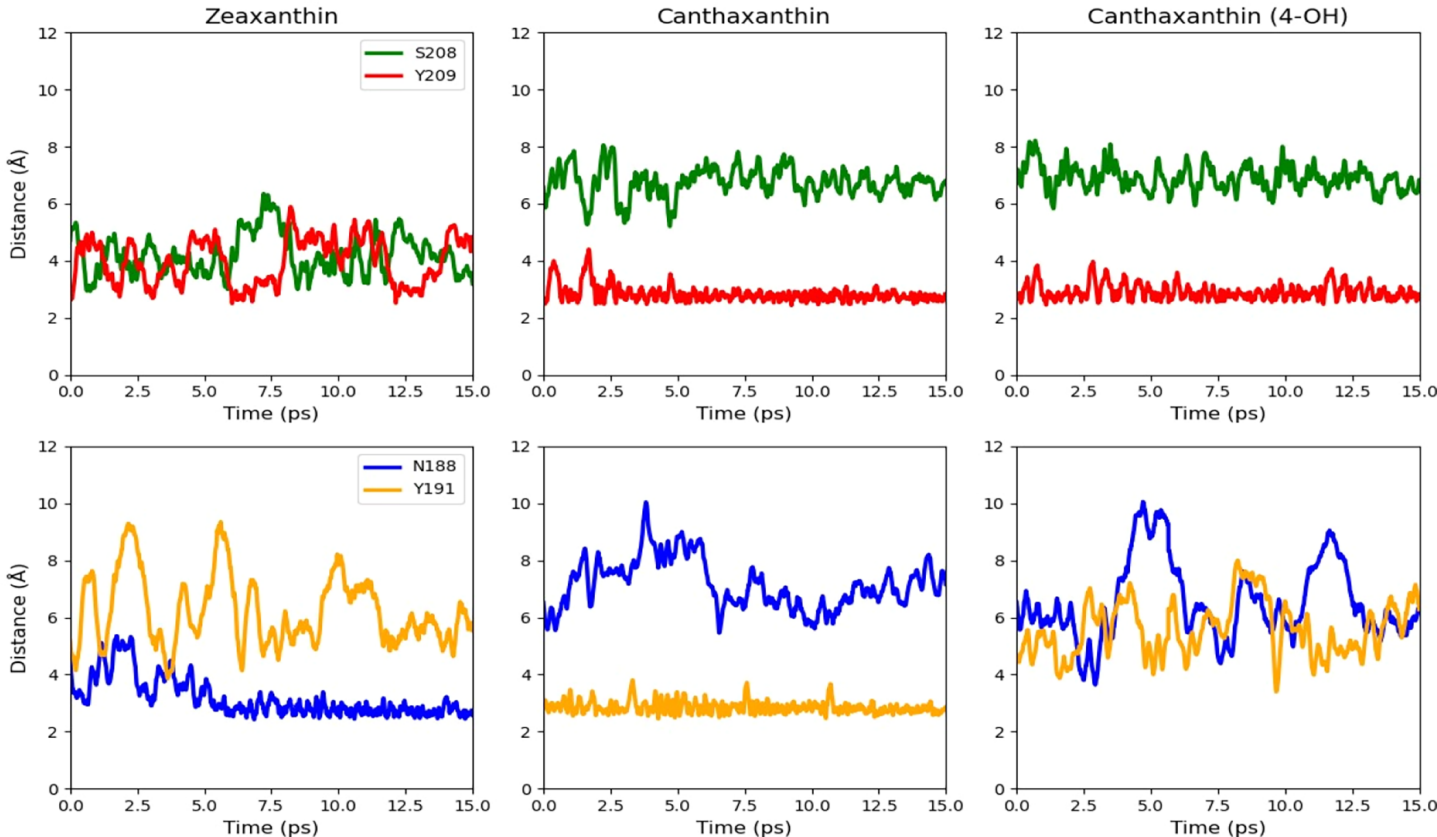
Distance between the carotenes and key residues during the hybrid QM/MM MD simulations. The top graphs show the interactions of ring A with residues Ser208 and Tyr209, while the bottom graphs show the distance from ring B to Asn188 and Tyr191.

## 3 Conclusions

Proton-pumping rhodopsins are employed by a variety of microorganisms that inhabit a broad range of sunlit environments. Following our recent study, which documented the potential binding of 3-hydroxylated lutein and zeaxanthin in XRs, we sought to identify other carotenoids that might serve as auxiliary antennae in XRs as well as the factors regulating their binding.

3-hydroxylated carotenoids (lutein and zeaxanthin) have been identified as the preferred supplementary chromophores for efficient excitation energy transfer to the Kin4B8 retinal, in addition to 2,3-dihydroxylated carotenoid nostoxanthin. In contrast, 4-ketocarotenoids (such as canthaxanthin and astaxanthin) are less effective as light-harvesting antennas, though the binding efficiency remains nearly similar. Notably, the presence of a hydroxy group at the C4 position on the ring impedes binding and energy transfer. Furthermore, the structure of the β-ionone ring plays a critical role, with substitution of both β-ionone rings by an ε-ring completely abolishing binding. Although the identity of the carotenoid bound to Kin4B8 in vivo remains unknown, our findings suggest that 3-hydroxylated carotenoids (lutein or zeaxanthin) exhibit superior antenna efficiency compared to ketocarotenoids. This implies that each rhodopsin may utilize a distinct selection of carotenoids.

The primary criterion for identifying a potential carotenoid binding site in a rhodopsin is the conserved glycine residue (Gly156 in XR, Gly153 in Kin4B8) rather than a bulky residue near the carotenoid ring. An additional important residue is conserved Tyr207 in XR (Tyr204 in Kin4B8), which may participate in a hydrogen bonding network that stabilizes the carotenoid in the binding site. A distinguishing feature between 4-keto and 3-hydroxy carotenoids may lie at the bottom of the binding site, which consists of polar residues for 3-hydroxy carotenoids (in Kin4B8, KR1, and EinA29G6) and relatively hydrophobic residues for 4-ketocarotenoids (in XR, GR). It appears that the proximity of a tyrosine residue to the keto group (such as Tyr207 in XR or Tyr204 in Kin4B8) may be a crucial factor in the binding of keto carotenoids to rhodopsins.

This study provides insights into the interaction of carotenoids with the representative rhodopsin Kin4B8 and offers valuable information about the structural determinants of carotenoid binding and specificity within the retinal protein binding site. Further studies using alternative rhodopsin models may yield additional insights, facilitating accurate prediction of the specific carotenoid selection by XRs and PRs.

## 4 Methods

### 4.1 Carotenoids

Lutein (PHR1699) and β-carotene (PHR1239) were purchased from Sigma-Aldrich, zeaxanthin (A132185) was purchased from AmBeed, and rhodoxanthin (SCPT-31617) was purchased from Shanghai Saikerui Biotechnology Co. Ltd. Salinixanthin was extracted from purified cell membranes of *S. ruber* expressing XR as described previously.^10^ Nostoxanthin was extracted from the bacterium *Sphingomonas glacialis* AAP5 using 100% methanol as previously described.^25^ The obtained extract was separated using a Shimadzu Prominence high-performance liquid chromatography system equipped with a semipreparative C18 column (250 × 8 mm, Kromasil 100-C18, 5um, Watrex Ltd., Czech Rep.), as previously described.^26^ Collected fractions were pooled and concentrated by using a rotary vacuum evaporator. Canthaxanthin was purified from the biomass of the microalgae *Haematococcus pluvialis*, supplied by Algamo, s.r.o., Mostek, Czech Republic. The dried biomass was subjected to solvent extraction with a 1:1 (*v*/*v*) mixture of ethanol and ethyl acetate. The resulting crude extract was fractionated using a two-step high-performance counter-current chromatography (HPCCC) protocol adapted from the method reported previously.^27^ For the first HPCCC separation step, a biphasic solvent system of *n*-heptane and acetonitrile (1:1, *v*/*v*) was used with the lower phase serving as the mobile phase and the upper phase as the stationary phase. A total of 120 mg of the crude extract was processed at a mobile phase flow rate of 1 mL/min. This procedure was carried out in duplicate, and the combined target fractions were concentrated by rotary evaporation under reduced pressure at 28 °C, yielding 2.05 mg of a canthaxanthin-enriched fraction. To further increase purity, a second HPCCC separation step was performed using a biphasic solvent system composed of *n*-heptane, acetone, ethanol, and water (1:1:1:1, *v*/*v*/*v*/*v*). In this step, the lower phase served as a mobile phase with a flow rate of 1 mL/min, while the upper phase remained stationary. The procedure yielded 1.85 mg of the canthaxanthin fraction. Subsequent purification was performed by gel permeation chromatography using Sephadex LH-20 as the stationary phase and 100% methanol as the mobile phase. The purified canthaxanthin fraction was then concentrated by rotary evaporation under reduced pressure at 28 °C, yielding 1.15 mg of high-purity canthaxanthin. Astaxanthin was isolated from the biomass of the microalgae *Haematococcus pluvialis*, sourced from Algamo, s.r.o., Mostek, Czech Republic. The dried biomass was subjected to extraction using a 1:1 (v/v) mixture of ethanol and ethyl acetate. The resulting algae extract was processed by a two-step high-performance countercurrent chromatography (HPCCC) procedure, based on modifications of the method described previously.^27^ For HPCCC, a two-phase solvent system comprising *n*-heptane and acetonitrile (1:1, v/v) was utilized, with the lower phase acting as the mobile phase and the upper phase as the stationary phase. In the first HPCCC separation step, 120 mg of the algae extract was processed at a mobile phase flow rate of 1 mL/min. This step was repeated twice, and the combined target fractions, after solvent removal by rotary evaporation under reduced pressure at 28 °C, yielded 1.94 mg of an astaxanthin-containing fraction. To enhance purity, a second HPCCC separation step was conducted at a reduced mobile phase flow rate of 0.5 mL/min, resulting in 1.18 mg of the astaxanthin fraction. Further purification of the astaxanthin fraction obtained from the second HPCCC step was accomplished by using gel permeation chromatography with Sephadex LH-20 gel and 100% methanol as the mobile phase. The resulting astaxanthin fraction was then concentrated using a rotary evaporator under reduced pressure at 28 °C, yielding 0.64 mg of pure astaxanthin.^27^ Lactucaxanthin was extracted from romaine lettuce plants. Three g of outer leaves of fresh romaine lettuce plants, acquired in a local store, were washed with distilled water and cut into 2 cm-long pieces after removing the midrib. The leaf pieces were frozen in liquid nitrogen and ground by manual grinding using a liquid-nitrogen-cooled mortar and pestle, and carotenoids were extracted with methanol and continuous grinding. The obtained extract, separated by centrifugation from the colorless cell debris, contained a mixture of carotenoids and was subsequently subjected to a solid-phase extraction (SPE) step with LiChrolut RP-18 Columns (Merck, Darmstad, Germany) and an acetone gradient (50–100%) as mobile phase to obtain individually purified lactucaxanthin, which was concentrated in a rotary vacuum centrifuge, lyophilized using a freeze-dryer and stored at–20 °C under N_2_ atmosphere.

### 4.2 Sample Preparation to Study Carotenoid Interaction with Rhodopsins

Concentrated stock solutions of lutein, zeaxanthin, and rhodoxanthin were prepared in DMSO; β-carotene, canthaxanthin, astaxanthin, and nostoxanthin were prepared in acetone; and lactucaxanthin was prepared in ethanol. A small excess molar equivalent of carotenoids was added to the protein (solubilized in 0.05% DDM, 0.3 M NaCl, and 0.05 M phosphate buffer at pH 7.5), followed by overnight incubation. The detailed procedure is as follows: For the carotenoid, we obtained the molar extinction coefficient in ethanol (or other organic solvents like DMSO or acetone, depending on the specific carotenoid’s solubility) from available literature reports. The molar concentration of the ethanolic stock solution of dissolved carotenoids was then determined by measuring the absorption spectra.

For the protein, the molar extinction coefficient (at the retinal absorption band) was calculated as follows. A DDM-solubilized protein solution (OD ∼ 0.2) in phosphate buffer (pH 7.5) was reacted with 200 mM hydroxylamine. The reaction was accelerated by illuminating the mixture with light >530 nm to fully convert the retinal protonated Schiff base to retinal oxime. The reaction’s progression was monitored by measuring the absorption spectra. The retinal absorption band at 565 nm shifted to ∼ 370 nm upon retinal oxime formation. Using the known molar extinction coefficient of retinal oxime in aqueous solution (33,600 M^–1^ cm^–1^) and the absorption of the produced retinal oxime, we determined the molar extinction coefficient and, consequently, the molar concentration of the protein.

For the preparation of the protein-carotenoid complex, a protein solution of the desired concentration was prepared in phosphate buffer, and carotenoids from a concentrated stock solution in ethanol, DMSO, or acetone were added in small volumes. A slight molar excess (∼1.5 times) of carotenoids was used to ensure saturation in protein-carotenoid binding.

Since both Kin4B8 and KR1 are His-tagged, the protein-carotenoid complex was purified using a Ni^2+^-NTA resin column to remove unbound carotenoids and subsequently used for spectroscopic studies, as previously described.^19^ Briefly, the sample was loaded onto a gravity-flow column pre-equilibrated with Ni-NTA resin (HisPur Ni-NTA resin, ThermoFisher Scientific) and buffer (50 mM phosphate buffer, pH 7.5, containing 0.05% DDM and 0.3 M NaCl). The sample was allowed to flow through the column, and the flow-through was discarded. To remove unbound carotenoids, the resin was washed with two resin bed volumes of wash buffer (50 mM MES, 300 mM NaCl, and 0.03% DDM, pH 6.0). The carotenoid-bound protein was then eluted using elution buffer (50 mM Tris-HCl, 300 mM NaCl, 250 mM imidazole, and 0.03% DDM, pH 7.5). The eluted sample was washed twice with a solution containing 100 mM NaCl and 0.02% DDM to remove imidazole and then concentrated using an Amicon Ultra centrifugal filter (10 kDa MWCO, Millipore). Finally, the purified protein was stored in 50 mM phosphate buffer (pH 7.5) containing 0.05% DDM and 0.3 M NaCl for further spectroscopic studies.

### 4.3 Reduction of the Carbonyl Group to Hydroxy in the Keto-Carotenoids

Canthaxanthin, astaxanthin, and salinixanthin contain a keto group at the C4 position and rhodoxanthin at the C3 position. The carotenoid was dissolved in ethanol (3–4 μM, 1.5 mL), and a freshly prepared ethanolic solution of NaBH_4_ was added to it.^28^ The progress of the reaction was monitored by taking the absorption spectra of the reaction mixture. The broad absorption band of the individual carotenoids was converted to a fine-structured band along with a blue shift in the absorption band position (Figures S3 and S4). To ensure fast and complete decomposition of NaBH_4_, methanol was added by ∼ 1.5 mL. The reactions took ∼ 15–30 min to complete, depending on the individual carotenoid. Following the evaporation of the alcoholic solvent, the obtained hydroxylated carotenoids were dissolved in the desired solvent to prepare the stock solution for further use.

### 4.4 Expression and Purification of Rhodopsin

Point mutations Y204A, S208A, and Y209F in Kin4B8 gene (cloned into a pBAD) were generated by following the NEB Q5 site-directed protocol (https://nebasechanger.neb.com/) by using the following primers: Y204A–5′- CAATCGTTGCAGCCATGGGAAG −3′, and 5′- GATAAAAACCCCAGGTTAACAGAATC −3′; S208A–5′- TGCTATGGGAGCATATGGATGGTTGG −3′, and 5′- TAAACGATTGGATAAAAACC −3′; Y209F–5′- TATGGGAAGCTTCGGATGGTTGG −3′, and 5′- GCATAAACGATTGGATAAAAAC −3′. Kin4B8 and Kin4B8 mutants were expressed following the procedure reported previously.^19^ Briefly, DH10b *E. coli* cells harboring a pBAD vector containing a Kin4B8 mutant were grown in LB supplemented with 50 μg mL^–1^ ampicillin at 130 rpm at 37 °C. OD at 600 nm reached 0.8, and expression was induced by 0.1% l-arabinose. The induced culture was grown in the presence of 20 μM *all*-*trans* retinal at 130 rpm overnight at 30 °C. KR1 was expressed according to the procedure reported previously.^19^ Briefly, BL21 (DE3) *E. coli* cells with the pET-21a-KR1 plasmid were grown on LB with 50 μg mL^–1^ ampicillin at 220 rpm and 37 °C. OD_600_ reaching 0.6, expression was induced using 0.5 mM isopropyl β-D-1-thiogalactopyranoside (IPTG) and supplemented with 20 μM all-*trans* retinal. The induced culture was grown at 130 rpm overnight at 30 °C. Kin4B8 mutants and KR1 protein-expressing cells were harvested by centrifugation for 15 min at 7000 rpm at 4 °C. The collected cells were resuspended in buffer S (50 mM MeS, 300 mM NaCl, 5 mM imidazole, 5 mM MgCl_2_; pH 6.0) containing 1% (w/v) *n*-dodecyl-β-D-maltoside (DDM) and lysed with lysozyme (0.1 mg/mL) overnight at 4 °C in the presence of DNase and a protease inhibitor. The protein, solubilized in DDM, was isolated by centrifugation (40 min, 18,000 rpm, 4 °C) and then loaded on a Ni^2+^-NTA resin (Thermo Fisher Scientific). Unspecific bound proteins were removed by washing with buffer W (50 mM MES, 300 mM NaCl, 50 mM imidazole, and 0.06% DDM; pH 6.0). His-tagged proteins were eluted with buffer E (50 mM Tris-HCl, 300 mM NaCl, 250 mM imidazole, and 0.06% DDM; pH 7.5). Eluted protein was washed using Amicon 30 kDa cutoff (Millipore) with the wash solution containing 100 mM NaCl, 10% glycerol, and 0.02% DDM.

### 4.5 Absorption Spectroscopy Measurements

All UV–Vis absorption spectra of the carotenoids in pure solvent/buffered DDM solution, purified DDM solubilized rhodopsins, and the rhodopsin-carotenoid complex in buffered DDM solution were measured with a Cary 8454 UV–vis spectrophotometer (Agilent Technologies). The concentrations of the protein and carotenoid were in the 2–6 μM range for absorption spectral measurements.

### 4.6 Circular Dichroism (CD) Spectroscopy Measurements

CD spectroscopy measurements of the pure carotene in buffered DDM solution, purified DDM-solubilized rhodopsins, and the rhodopsin-carotenoid complex were performed with a Chirascan CD spectrometer (Applied Photophysics). All the spectra were recorded with 2.1 nm bandwidth resolution at 1 nm intervals using a quartz cell of a 1 cm path length.

### 4.7 Fluorescence Spectroscopy Measurements

Fluorescence emission and excitation spectral measurements were performed on a Jobin Yvon-Spex Fluorolog-3. The spectrofluorometer consists of a 450W Xe-lamp as the light source, a double-grating monochromator, and a Hamamatsu photomultiplier tube detector (R928P). Both the emission and excitation channel slit widths were mostly kept at 8 or 10 nm. OD of the samples for fluorescence measurements was maintained within 0.1 with respect to the maximum absorption of the rhodopsin. However, the obtained spectral profiles were further corrected for the internal absorption effect as *F*_*i*_ = *F*_obs_0.10^(*A*ex+*A*em)/2^, where *F*_*i*_ and *F*_obs_ are the ideal and measured fluorescence intensity, respectively; and *A*_ex_ and *A*_em_ are the absorbances at the excitation and emission wavelengths, respectively.^29^ Excitation spectra were sampled at 720 nm in order to avoid strong Raman bands that mask the retinal fluorescence. Fluorescence excitation spectra were scaled to the respective retinal absorption maxima for the calculation of excitation energy transfer efficiency of the respective donor–acceptor pairs.^19^ Using the fluorescence excitation spectral profile and the corresponding absorption spectral profile of the rhodopsin-carotenoid complex, the quantum efficiency of excitation energy transfer (from the carotenoid antenna to the retinal chromophore) was estimated with the following equation: Exc­(λ) *=*(1–10^–A^) × (*A*_r_ + ϕ*A*_c_)/*A*. Where Exc­(λ) and *A* (=*A*_r_ + *A*_c_) are the excitation spectrum and the absorbance of the complex, respectively; *A*_c_ and *A*_r_ are the absorption spectra of bound carotenoid and the retinal component, respectively; ϕ is the quantum efficiency of energy transfer.^7^

### 4.8 Computational Methodology

The crystal structure of Kin4B8 was used as the initial starting point for all subsequent calculations. This experimental structure originally contained zeaxanthin. Structures with canthaxanthin (4-keto) and canthaxanthin (4-hydroxy) were built by making the required structural modifications to the zeaxanthin chromophore using the Molefracture program in VMD.^30^ The resulting structures were then optimized using the hybrid quantum mechanics/molecular mechanics (QM/MM) scheme in Orca 5.0.3.^31,32^ In this method, the MM region was treated with the AMBER force field, while the QM region was treated using B3LYP/Def2-SVP.^33,34^ During the optimization, side chains within 5 Å of the carotenoid and retinal protonated Schiff base were relaxed, while the protein backbone and remaining side chains were frozen.

To observe the binding dynamics of the various carotenoids with the protein, the models were placed in a lipid bilayer of POPC molecules, and both sides of the bilayer were solvated with water and ions using the CHARMM-GUI Web site.^35^ Multiscale QM/MM Molecular dynamics analyses were performed at 300 K over 15 ps to model the binding dynamics of the carotenoids to the proteins. These calculations were performed using the Terachem 1.9.3 computational package linked to the AMBER force field through a modified version of the Chemshell TCL package.^36^ Before dynamics was performed, the proteins were reoptimized inside the lipid bilayer while allowing side chains/lipids/waters, and ions within 10 Å of the carotenoid to be relaxed. Following the optimization, QM/MM dynamics were performed, and the distance between the oxygen of the hydroxy/keto groups of Rings-A, B to residues Ser208, Tyr209 (Ring-A) and Tyr191, Asn188 (Ring-B) was analyzed (see Figure 10).

## Supplementary Material



## Notes

Interaction of canthaxanthin with Kin4B8; interaction of astaxanthin (4-hydroxy) with Kin4B8; interaction of rhodoxanthin (3-hydroxy); comparison of the absorption spectrum of all the hydroxylated carotenoids in ethanol; comparison of absorption spectrum of all the ketolated carotenoids in ethanol; interaction of canthaxanthin with KR1; interaction of astaxanthin with KR1; comparative interaction between 3-hydroxy carotenoid-Kin4B8 and 4-ketocarotene-Kin4B8; comparison of the β-ring of zeaxanthin and the ε-ring of lutein; molecular structure of zeaxanthin in s-cis conformation, and retinal in s-trans planner conformation; estimation of binding efficiency; and bond and torsional angles (in degrees) of the rings of the carotenoids zeaxanthin and lutein (PDF)

## ○

Experimental Biophysics, Department of Biology, Humboldt-Universität zu Berlin, Berlin 10115, Germany

## ◆

Institute of Environmental Engineering, ETH, Zurich 8093 Switzerland.

## ¶

Department of Physics, TU Dortmund University, 44227 Dortmund, Germany.
