# Supplementary material for: Selective Choice of the Efficient Carotenoid Antenna by a Xanthorhodopsin: Controlling Factors for Binding and Excitation Energy Transfer
Source: JACS Au. 2025 Jun 26;5(7):3070–81. doi: 10.1021/jacsau.4c01243 (PMC12308411; doi:10.1021/jacsau.4c01243)
Supplement: Supplementary file 1 [file au4c01243_si_001.pdf]

Supplementary information

Selective choice of the efficient carotenoid antenna by a xanthorhodopsin: controlling factors for binding and excitation energy transfer

Ishita Das[1,§,†], Ariel Chazan[2,§,‡], Jonathan R. Church[3], Shirley Larom[2], Rosa León[4], Patricia Gómez-Villegas[4], Daniela Bárcenas-Pérez[5,6], José Cheel[5], Michal Koblížek[5], Oded Béjà[2,7,*], Igor Schapiro[3,‖,*], and Mordechai Sheves[1,*]

[1]*Department of Molecular Chemistry and Materials Science, Weizmann Institute of Science, Rehovot 7610001, Israel*

[2]*Faculty of Biology, Technion-Israel Institute of Technology, Haifa 3200003, Israel*

[3]*Fritz Haber Center for Molecular Dynamics Research Institute of Chemistry, The Hebrew University of Jerusalem, Jerusalem 9190401, Israel*

[4]*Laboratory of Biochemistry and Molecular Biology, Faculty of Experimental Sciences, Marine International Campus of Excellence (CEIMAR), University of Huelva, Huelva 21071, Spain*

[5]*Centre Algatech, Institute of Microbiology, Novohradská, Třeboň 37981, Czech Republic*

[6]*Faculty of Science, University of South Bohemia, Branišovská 1760, 370 05 České Budějovice, Czech Republic*

[7]*The Nancy and Stephen Grand Technion Energy Program (GTEP), Technion-Israel Institute of Technology, Haifa 3200003, Israel*

[§]*These authors contributed equally: Ishita Das, Ariel Chazan*

**To whom correspondence should be addressed: Igor Schapiro (igor.schapiro@mail.huji.ac.il), Oded Beja beja@technion.ac.il, Mordechai Sheves (mudi.sheves@weizmann.ac.il)*

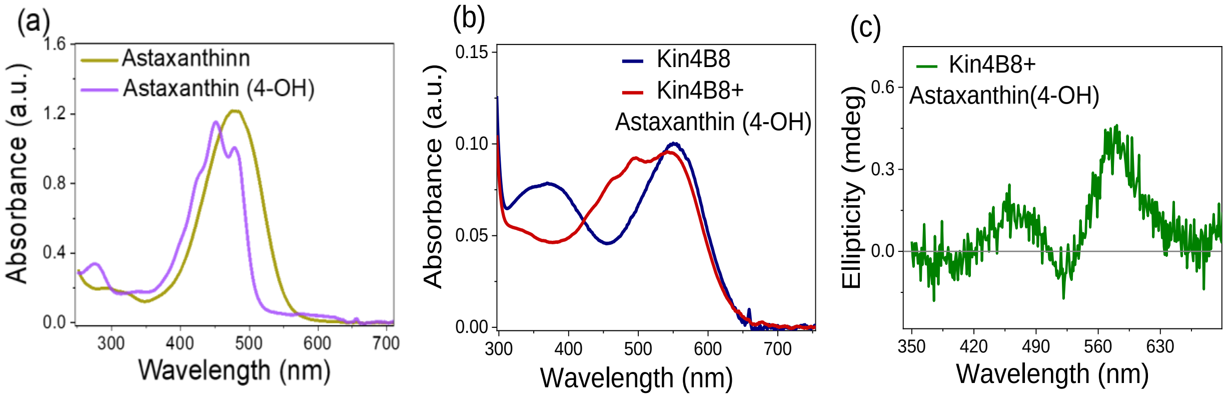

Figure S1. (a) Comparative absorption spectra of astaxanthin and astaxanthin (4-hydroxy) in ethanol. Interaction of astaxanthin (4-hydroxy) with Kin4B8 studied with (b) absorption spectra (c) CD spectrum.

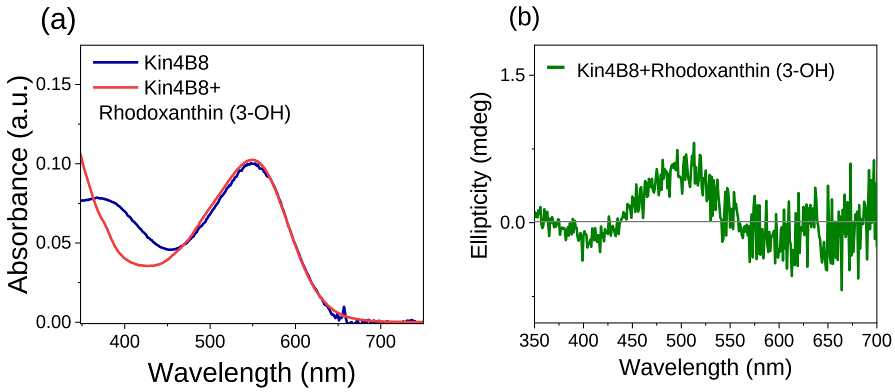

Figure S2. Interaction of rhodoxanthin (3-hydroxy) with Kin4B8 studied with (a) absorption spectrum (b) CD spectrum.

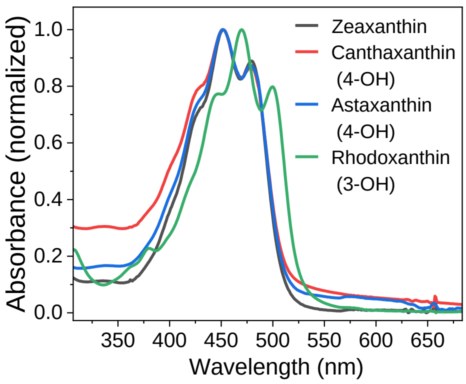

Figure S3. Comparison of absorption spectra (normalized) of the hydroxycarotenoids in ethanol.

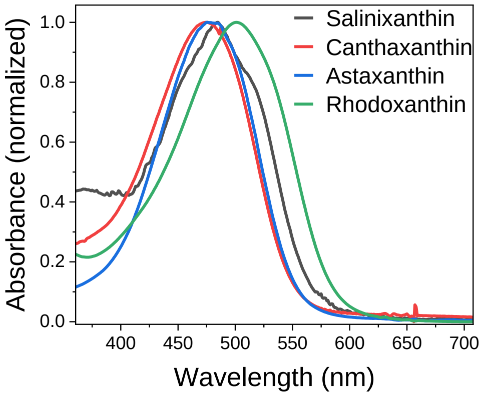

Figure S4. Comparison of absorption spectra (normalized) of the ketocarotenoids in ethanol.

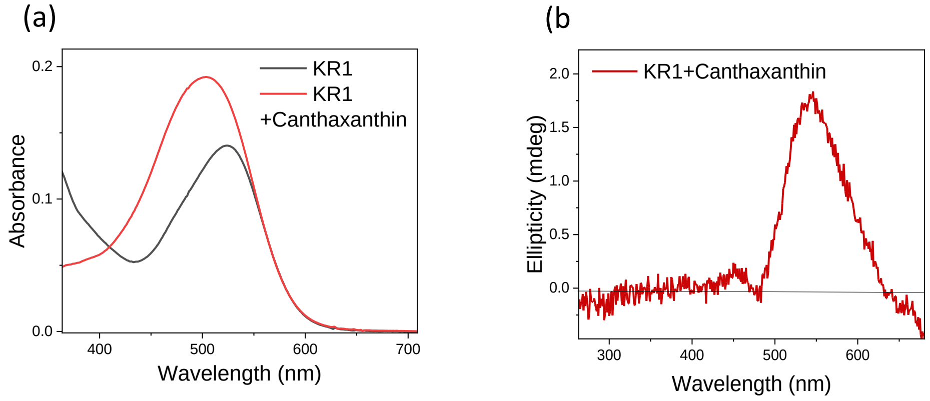

Figure S5. Interaction of canthaxanthin with KR1 studies with (a) Absorption spectra (b) CD spectra.

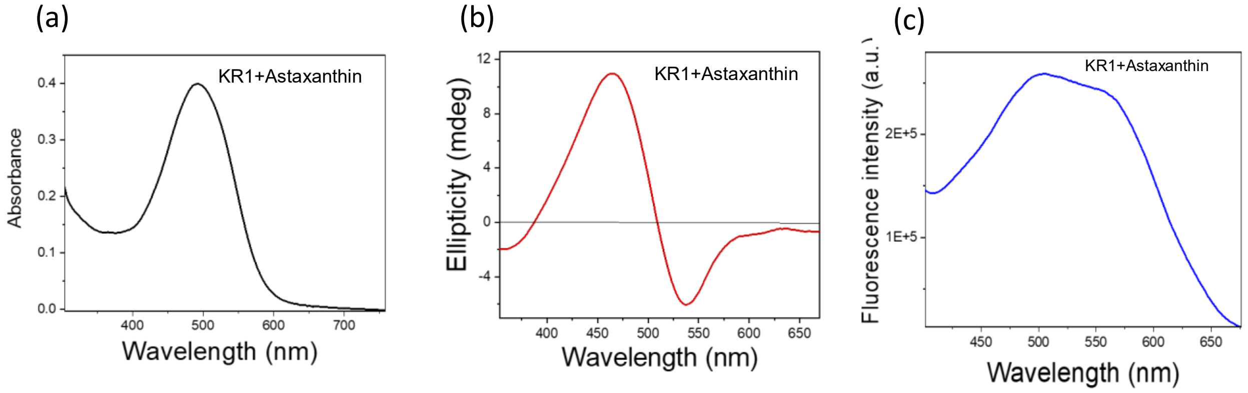

Figure S6. Interaction of astaxanthin with KR1 studied with (a) absorption spectra (b) CD spectra (c) fluorescence excitation spectra.

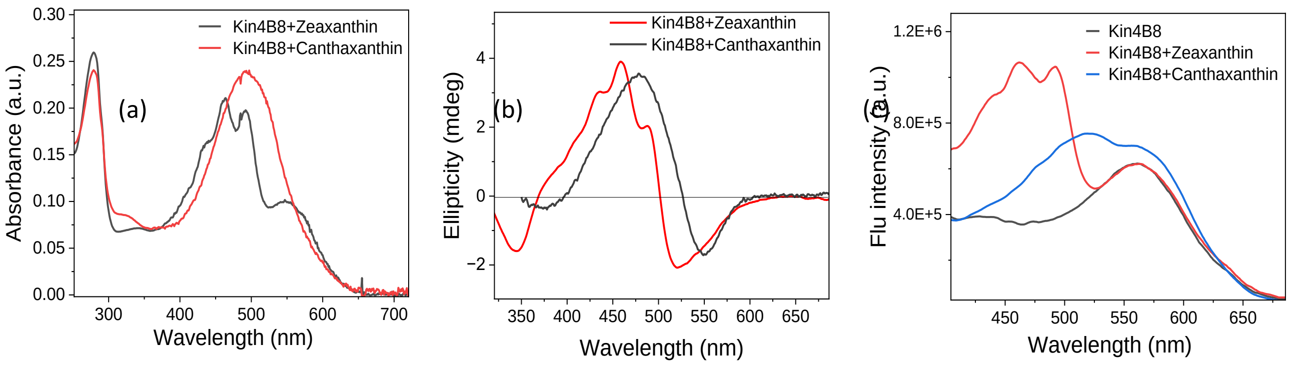

Figure S7. Comparison in the interaction between 3-hydroxy carotenoid (zeaxanthin)-Kin4B8 and 4-ketocarotene (canthaxanthin)-Kin4B8 studied with (a) absorption spectra (b) CD (c) fluorescence excitation spectroscopy.

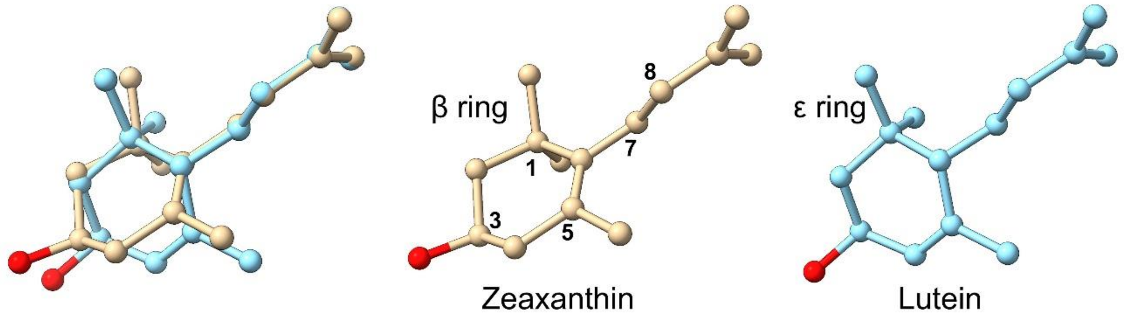

Figure S8. Comparison of the β-ring of zeaxanthin and the ε-ring of lutein.

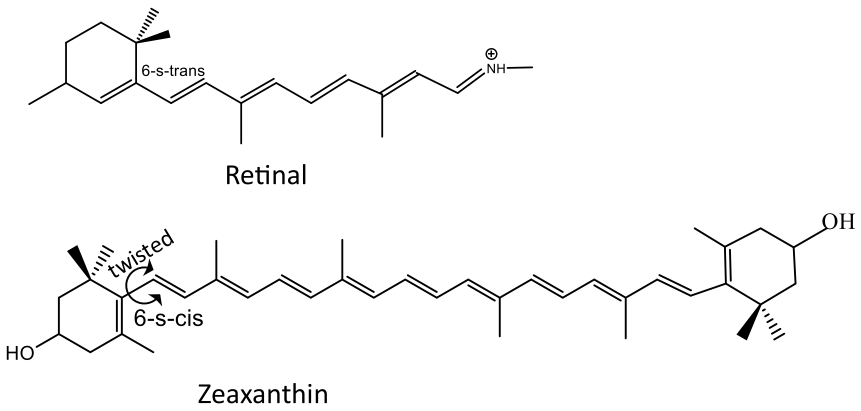

Scheme S1. Molecular structure of zeaxanthin in 6-s-cis conformation, and retinal in 6-s-trans planar conformation

Table S1. Molar ratio of the carotenoid/protein in the complex as obtained experimentally, and estimated binding efficiency

Complex	OD_{car}	OD_{Kin4B8}	Observed OD_{car}/OD_{Kin4B8}	Expected OD_{car}/OD_{Kin4B8} as per the molar absorptivity (for 1:1 molar ratio)	Binding percentage (%)
**Kin4B8-Zeaxanthin**	0.22	0.1	2.2	2.5	88
**Kin4B8-Lutein**	0.22	0.1	2.2	2.5	88
**Kin4B8-Canthaxanthin**	0.18	0.1	1.8	2.2	82
**Kin4B8-Astaxanthin**	0.18	0.1	1.8	2.2	82

*The molar absorptivity (ε) of the carotenoids has been used as follows. Zeaxanthin (ethanol): 145000 M^{-1} cm^{-1}, lutein (ethanol): 145000 M^{-1} cm^{-1}, canthaxanthin (petroleum ether): 124000 M^{-1} cm^{-1}, astaxanthin (hexane): 125000 M^{-1} cm^{-1}; and Kin4B8 (aqueous buffer): 56320 M^{-1} cm^{-1}.

Table S2. Bond and torsional angles (in degrees) of the rings of the carotenoids and the magnitude of difference between them.

**Angle**	**Zeaxanthin** (β-ring)	**Lutein** (ε-ring)	**|Diff.|**
C1-C2-C3	112.6	113	0.4
C2-C3-C4	109.6	110.5	0.9
C3-C4-C5	114.6	124.7	10.1
C4-C5-C6	122.4	122.2	0.2
C5-C6-C1	122.4	113	9.4
**Torsion**			
C1-C2-C3-C4	60.9	47.8	13.1
C2-C3-C4-C5	-43.6	-16.3	27.3
C3-C4-C5-C6	16.1	0.0	16.1
C4-C5-C6-C1	-3.3	-14.2	10.9
C5-C6-C1-C2	18.7	43	24.3
C6-C1-C2-C3	-47.6	-61.9	14.3
C8-C7-C6-C5	-56.4	-124.4	68
C8-C7-C6-C1	126.5	108	18.5
